# Cerebello‐Prefrontal Connectivity Underlying Cognitive Dysfunction in Spinocerebellar Ataxia Type 2

**DOI:** 10.1002/acn3.70028

**Published:** 2025-04-03

**Authors:** Ami Kumar, Ruo‐Yah Lai, Zena Fadel, Yicheng Lin, Pia Parekh, Rachel Griep, Ming‐Kai Pan, Sheng‐Han Kuo

**Affiliations:** ^1^ Department of Neurology, College of Physicians and Surgeons Columbia University New York New York USA; ^2^ Initiative for Columbia Ataxia and Tremor Columbia University New York New York USA; ^3^ Teachers College Columbia University New York New York USA; ^4^ Taipei Municipal Gan‐Dau Hospital, Taipei Veterans General Hospital Branch Taipei Taiwan; ^5^ Cerebellar Research Center National Taiwan University Hospital, Yun‐Lin Branch Yun‐Lin Taiwan; ^6^ Department and Graduate Institute of Pharmacology National Taiwan University College of Medicine Taipei Taiwan; ^7^ Department of Medical Research National Taiwan University Hospital Taipei Taiwan; ^8^ Institute of Biomedical Sciences Academia Sinica Taipei Taiwan

**Keywords:** cerebellum, cognition, electroencephalogram (EEG), functional connectivity, spinocerebellar ataxia type 2 (SCA2)

## Abstract

**Objective:**

Spinocerebellar ataxia type 2 (SCA2) is a hereditary cerebellar degenerative disorder, with motor and cognitive symptoms. The constellation of cognitive symptoms due to cerebellar degeneration is named cerebellar cognitive affective syndrome (CCAS), which has increasingly been recognized to profoundly impact patients' quality of life; however, the brain circuits underlying these cognitive dysfunctions remain elusive.

**Methods:**

We utilized a novel technique, cerebello‐cortical electroencephalogram (EEG), to investigate the resting‐state functional connectivity in different frequency domains in 12 SCA2 patients and 24 age‐matched controls. Given that the prefrontal cortex is strongly connected to the cerebellum, we studied the EEG connectivity between the cerebellum and the prefrontal cortex. We also conducted correlation analyses to explore the association between this connectivity and the severity of cognitive dysfunction, determined by CCAS scores.

**Results:**

Source‐space spectral analysis differences between SCA2 patients and controls were observed in the cerebellum at the delta, theta, and beta frequencies. Functional connectivity between the posterior cerebellum and the prefrontal cortex revealed decreased theta and increased beta connectivity in SCA2 patients, with no differences in delta connectivity. Increased beta connectivity was unique to the prefrontal regions, not seen in the connectivity to the primary motor cortex or mid‐temporal lobe. Interestingly, this beta connectivity correlated with CCAS scores in SCA2 patients.

**Conclusion:**

Our findings demonstrated that SCA2 patients have an increase in beta cerebello‐prefrontal connectivity that correlates with cognitive performance. These findings suggest cerebello‐cortical EEG could track circuit dysfunction underlying cognitive symptoms in SCA2, paving the way for developing targeted neuromodulation therapeutics.

## 1 Introduction

Spinocerebellar ataxia type 2 (SCA2) is a hereditary cerebellar neurodegenerative disorder characterized by progressive impairment of motor (gait, incoordination, balance) and cognitive deficits [1, 2]. The cognitive deficits associated with cerebellar degeneration are a cluster of symptoms known as cerebellar cognitive affective syndrome (CCAS) [3], which encompasses impairment in executive function, visuospatial processing, and affective regulation. CCAS significantly challenges daily functioning and substantially diminishes the overall quality of life for individuals with cerebellar degeneration [4, 5]. Despite the growing recognition of CCAS, the brain circuits underlying these cognitive symptoms remain poorly understood, presenting a major hurdle toward therapeutic development.

Over the years, resting‐state electroencephalogram (EEG) has been a focus of exploring intrinsic brain activity and functional brain organization [6, 7]. Multiple studies demonstrate that resting‐state connectivity, measured by EEG, serves as a valuable means to explore whole‐brain circuit mechanisms relevant to various neuropsychiatric disorders [8, 9, 10]. This measure allows for an in‐depth understanding of how different frequency ranges contribute to the coordination and integration of neural processing within specific brain circuits. Along this line, surface resting‐state EEG over the cerebral cortex has provided tremendous insights into circuit dysfunction in schizophrenia, depression, epilepsy, and Alzheimer's disease [8, 11, 12, 13]; however, EEG has not been widely adopted to study circuit dysfunction of cerebellar degenerative disorders [14]. Our group [15, 16, 17] and others [18, 19, 20] have recently discovered that the cerebellum exhibits unique electrophysiological features that can be captured by the surface EEG in essential tremor and Parkinson's disease, respectively. Therefore, we applied the cerebellar EEG technique, along with cerebral EEG, to probe the circuit dysfunction of SCA2, one of the common SCAs associated with prominent cognitive and emotional symptoms.

While the cerebellum forms connections with multiple regions of the cerebral cortex, the cerebello‐prefrontal connectivity stands out as the most prominent, evolutionarily conserved pathway [21, 22]. This connectivity plays a central role in cognitive processes such as working memory, decision‐making, and executive function [23], making it a critical area of study. The prefrontal cortex is integral to higher‐order cognitive functions, while the cerebellum is increasingly recognized for its contributions to cognition beyond motor control. SCA2 patients have prominent cognitive symptoms related to cerebellar dysfunction; therefore, we chose to study SCA2 as the disease model to probe the circuit for cerebellum‐related cognitive dysfunction. SCA12, SCA17, and dentatorubral–pallidoluysian atrophy, which are also associated with significant cognitive impairments, were not included as part of the target group due to limited case numbers. We hypothesized that cerebello‐prefrontal connectivity exhibits frequency‐specific alterations in SCA2 patients, reflecting disruptions in the underlying neural circuits. By focusing on this connectivity, we aim to investigate the pathophysiology linking cerebellar dysfunction to cognitive impairment, providing insights into the neural substrates of these deficits and setting the foundation to identify potential targets for therapeutic interventions.

## 2 Methods

### 2.1 Subjects

We recruited SCA2 patients (*n* = 12) from the Ataxia Center of Excellence at Columbia University Irving Medical Center. The diagnosis of SCA2 was confirmed by the genetic tests identifying pathological repeat expansions of *ATXN2* and the presence of symptoms of cerebellar ataxia. We recruited age‐matched controls (*n* = 24) from friends or spouses of patients without a history of neurological disorders. To ensure the robustness and reliability of comparisons and account for variability, we included a 1:2 ratio of patients to controls. None of the controls have a diagnosis of neuropsychiatric disorders. The inclusion criteria for SCA2 patients are (1) aged 18–75, (2) no prior history of traumatic brain injury or other neurological diagnosis, and (3) able to perform cognitive rating scales. The severity of ataxia symptoms was assessed by the Scale for the Assessment and Rating of Ataxia (SARA) [24], a clinical tool that evaluates gait, stance, coordination, and speech to measure ataxia severity. Cognition was measured using the CCAS scale [3], which assesses cognitive deficits associated with cerebellar dysfunction, such as executive function, language, visuospatial processing, and affect regulation. Together, these two scales offer a detailed evaluation of motor and cognitive impairments in SCA2 patients. All participants signed the informed consent approved by the Institutional Review Board of Columbia University.

### 2.2 Cerebello‐Cortical EEG Recording

Resting‐state cerebello‐cortical EEGs were recorded for all SCA2 patients and controls using the BrainVision EEG recording system, which included an actiChamp amplifier and a custom actiCap (Brain Vision LLC, Morrisville, NC, USA). The custom actiCap consisted of 32 cortical electrodes and 18 cerebellar electrodes. The cerebellar electrodes were positioned laterally to the inion and subinion over the posterior fossa, corresponding to the left and right cerebellar lobules. The caps used were based on standard male and female head sizes to ensure a tight fit. The reference electrode was located between Fz and Cz, and the sampling rate was 500 Hz. Resting‐state EEG was recorded for 2 min, consistent with standard practices in previous studies [25, 26, 27], from participants sitting quietly, with their eyes open, looking straight ahead, to avoid the intrusion of alpha rhythm from the occipital cortex. Participants were further instructed to remain still and inhibit eye movements.

### 2.3 Data Analysis

#### 2.3.1 Preprocessing

Data analysis was performed using the Brainstorm open‐source toolbox [28]. The resting‐state EEG data was band‐pass filtered between the range of 1–35 Hz, and the channels were re‐referenced to the common average reference. From the continuous 2‐min dataset of each participant, artifact correction was done. To remove artifacts due to eye blinks, muscle movements, and noisy channels, we applied the signal space projection (SSP) [29] approach. SSP is a method for artifact correction that identifies and models artifacts as specific subspaces in the data. A projection matrix is then constructed to remove the contributions of these artifact subspaces while preserving the neural signals of interest. By effectively filtering out unwanted components, SSP minimizes artifacts in the data without significantly compromising the underlying brain activity. After artifact removal, the continuous EEG data for each participant was segmented, and 60 clean epochs of 1 s each were selected per participant, resulting in 1440 epochs for controls and 720 epochs for SCA2 patients.

#### 2.3.2 Source‐Space Spectral Analysis and Functional Connectivity

To calculate the connectivity between the cerebellum and prefrontal cortex, we first calculated the source activity underlying resting‐state EEG data. We constructed the head model using standard T1‐weighted MRI scans, employing the boundary element method to create a volumetric realistic representation of the head, involving segmentation into distinct layers including scalp, skull, and brain [30]. A source grid with a resolution of 5 mm comprising 15,235 points was then computed to represent potential neural sources, adapted to encompass the cerebellum, enhancing anatomical accuracy. Sources were estimated from EEG data, using the standardized low‐resolution brain electromagnetic tomography (sLORETA) [31] method. sLORETA utilizes the minimum norm imaging approach to estimate the distribution of neural activity within the brain based on measured scalp electric potentials. Subsequently, source‐space spectral power was estimated to identify frequency ranges that showed differences between SCA2 patients and controls within the cerebellum. Spectral power for all source time series from each subject was computed using Welch's method, employing a window length of 1 s with 50% overlap. The analysis targeted frequencies within the delta (1–3 Hz), theta (4–7 Hz), alpha (8–12 Hz), and beta (13–30 Hz) bands. Spectral power values at each frequency bin were normalized relative to the total power across the complete frequency spectrum to ensure standardization across different brain regions and participants. Based on the observed spectral power differences between SCA2 patients and controls, the cerebellar region of interests (ROI) and the frequency bands of interest were selected for further analysis. Furthermore, the cortex and the cerebellum were parcellated into distinct ROIs using the automated anatomical labeling 3 (AAL3) [32].

We calculated functional connectivity between the ROIs using the imaginary coherence method. The imaginary coherence method, employed for functional connectivity analysis, quantifies the phase synchrony between neural oscillations without being influenced by volume conduction effects, providing accurate measures of functional connectivity at the source level [33].

### 2.4 Statistical Analysis

To evaluate the statistical significance of differences between groups, we employed a nonparametric permutation *t*‐test [34]. A Monte Carlo approach was used to estimate the significance probability, involving 1000 random permutations of the data. The permutation test avoids assumptions about the data being normally distributed. The resulting *p* values were adjusted to address the multiple comparison problem using the false discovery rate correction method, which effectively controls the expected proportion of false positives while preserving statistical power. Two‐tailed *p* values of < 0.05 were considered statistically significant. Further, Pearson's correlation was estimated to calculate the correlation between the functional connectivity scores and CCAS scores or SARA scores for SCA2 patients.

## 3 Results

### 3.1 Patient Demographics

SCA2 patients have a comparable age to controls (SCA2: 47.0 ± 12.5 years old vs. control: 48.0 ± 16.2 years old). The average age of onset for SCA2 patients in this group was 36.0 ± 11.7 years old, with an average disease duration of 13.8 ± 8.2 years. These SCA2 patients had an average number of 40.0 ± 2.4 pathological CAG repeats in *ATXN2*. The SARA score for these patients was 14.2 ± 4.4, and the total CCAS score was 79.8 ± 11.9, indicative of their motor and cognitive dysfunction, respectively (Table 1).

**TABLE 1:** Demographic data of subjects.

	Control	SCA2	*p*
*n*	24	12	
Age (years)	48.0 ± 16.2	47.0 ± 12.5	0.736^a^
Age of onset (years)	N/A	36.0 ± 11.7	N/A
Disease duration (years)	N/A	13.8 ± 8.2	N/A
Gender			
Men	45.8%	41.6%	0.812^b^
Women	54.1%	58.3%	
CAG repeats	N/A	40.0 ± 2.4	N/A
Total SARA	N/A	14.2 ± 4.4	N/A
Total CCAS	N/A	79.8 ± 11.9	N/A

*Note:* Values represent mean ± standard deviation.

Abbreviations: CCAS, Cerebellar Cognitive Affective/Schmahmann Syndrome Scale; N/A, not applicable; SARA, Scale for the Assessment and Rating of Ataxia.

^a^ Welsh's *t*‐test.

^b^ Chi‐squared test.

### 3.2 ROIs for Functional Connectivity

**FIGURE 1:**
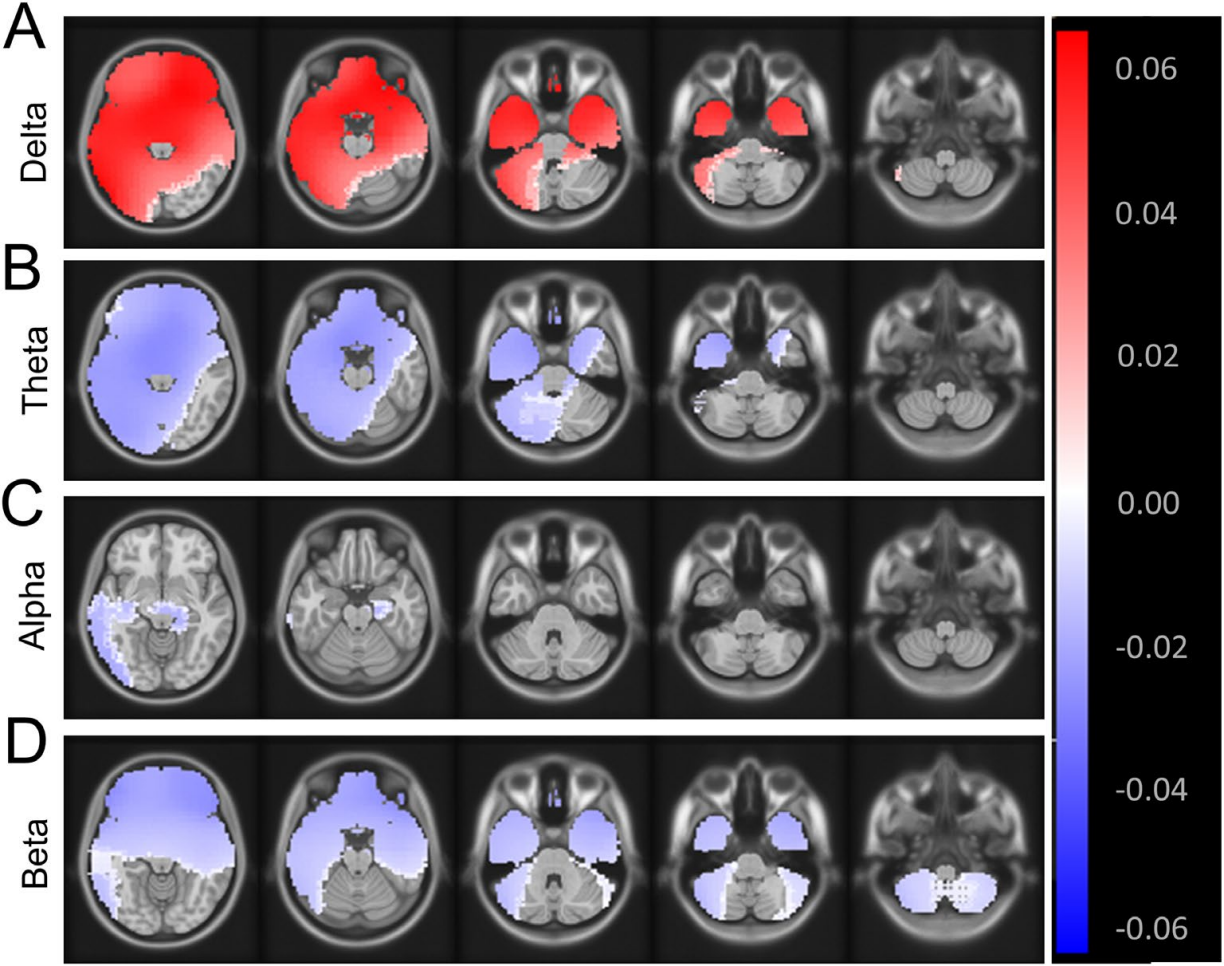
Source‐space spectral analysis. (A–D) Source‐space spectral power significant difference (*p* < 0.01), as observed for delta, theta, and beta frequency ranges for the cerebellum. The figure demonstrates the statistical threshold from the *t*‐test applied to the spectral power differences between SCA2 patients and controls. SCA2 patients have a decrease in spectral power in delta range while an increase in theta and beta frequency ranges for the cerebellum, as well as other cortical regions.

We performed the source‐space spectral analysis in the cerebellum and found significant differences in delta, theta, and beta frequency ranges (*p* < 0.01) between SCA2 patients and controls, with SCA2 patients exhibiting a decrease in delta power and an increase in theta and beta frequency ranges (Figure 1A,B,D). We did not observe any differences in the alpha range (Figure 1C). We further extracted the spectral power from the posterior cerebellar regions and found that the left Crus I region demonstrated the strongest differences consistently over delta (*p* = 0.007), theta (*p* = 0.001), and beta power (*p* = 0.001) between SCA2 patients and controls. Given our consistent observation of Crus I in the spectral analyses and its known role in cognition, we thus selected Crus I for further functional connectivity analysis. Crus I has strong anatomical connections to the prefrontal cortex, including the superior frontal gyrus and orbitofrontal cortex via the thalamus, and these parts of the cerebral cortex are critical for higher cognitive processes [35, 36]; therefore, we next studied the cerebello‐prefrontal connectivity between Crus I and the superior frontal gyrus and the orbitofrontal cortex, respectively. This combined approach ensured that the cerebellar ROI reflected observed group differences, while prefrontal ROIs were informed by their functional relevance in cognitive networks. These ROIs were extracted from the AAL3 parcellation of the cerebellum and cerebral cortex. Since the source‐space spectral analysis differences were most prominent in delta, theta, and beta frequency ranges, we focused on calculating the functional connectivity between the ROIs for these ranges.

### 3.3 Functional Connectivity

**FIGURE 2:**
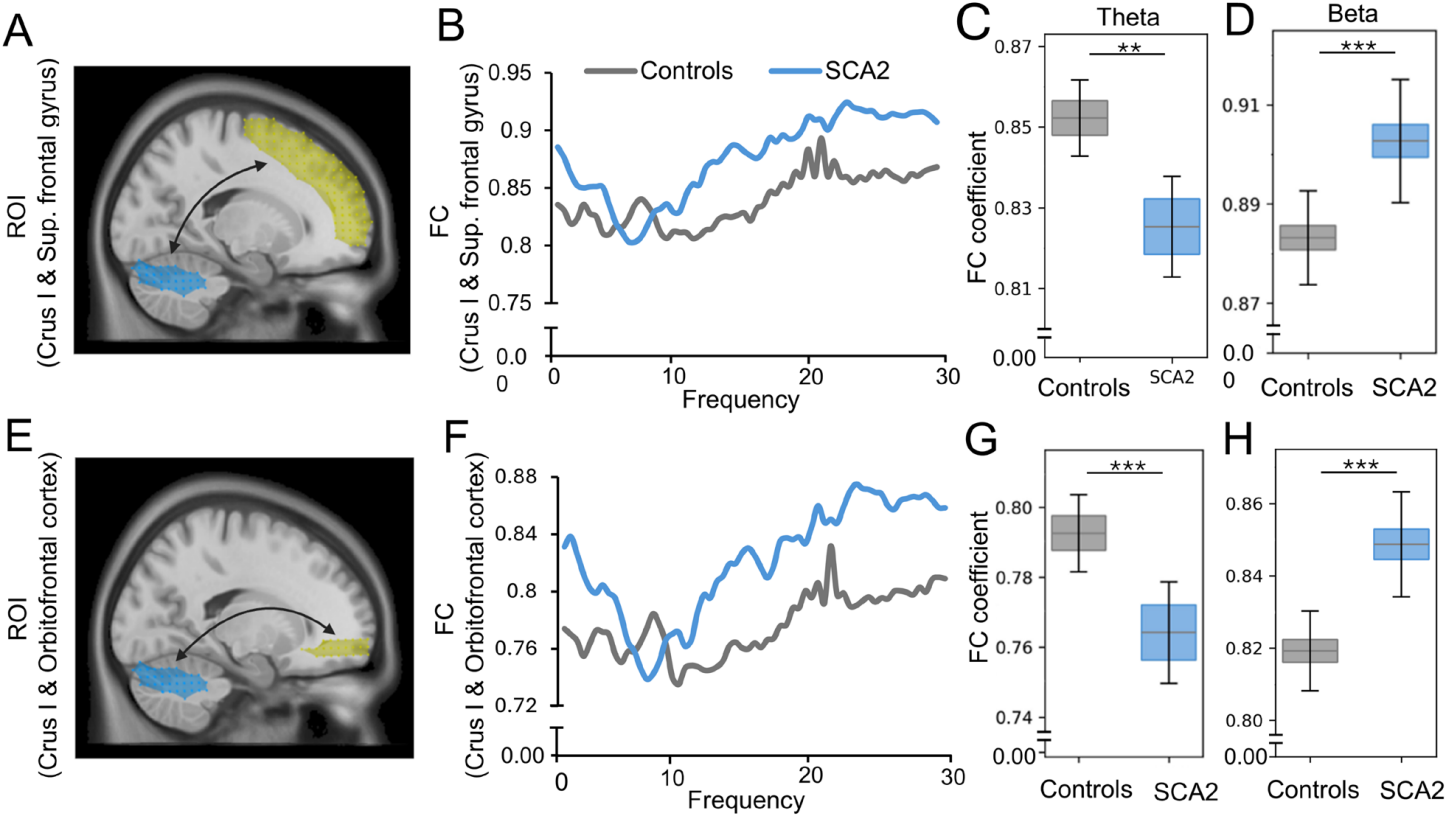
Functional connectivity between Crus I and prefrontal cortex. (A) Functional connectivity (FC) between Crus I and superior frontal gyrus. (B) Averaged FC spectral diagram between controls (*n* = 24) and SCA2 patients (*n* = 12) demonstrating (C) a significant decrease of FC in the theta frequency range and (D) an increase of FC in beta frequency range for SCA2 patients as compared to controls. (E–H) FC between Crus I and orbitofrontal cortex. FC coefficient (*y*‐axis) demonstrates the mean and standard error from the functional connectivity values calculated for the respective cerebellar and prefrontal ROIs between controls and patients. **p < 0.01, ***p < 0.001. ROI, region of interest; Sup, superior.

For the functional connectivity between Crus I (157 vertices, volume 13.04 cm^3^) and the superior frontal gyrus (317 vertices, volume 33.44 cm^3^) (Figure 2A), we found that SCA2 patients demonstrated a reduction in the theta frequency range (*p* = 0.001, Figure 2B,C) and an increase in the beta frequency range (*p* < 0.001, Figure 2B,D) as compared to controls. Similarly, for the connectivity between Crus I and the orbitofrontal cortex (58 vertices, volume 2.88 cm^3^), SCA2 patients also exhibited a reduction in theta frequency (*p* < 0.001, Figure 2E–G) and an increase in beta frequency connectivity (*p* < 0.001, Figure 2E,F,H). We did not observe differences in such connectivities in the delta frequency range in SCA2 patients versus controls (Figure S1A,B).

**FIGURE 3:**
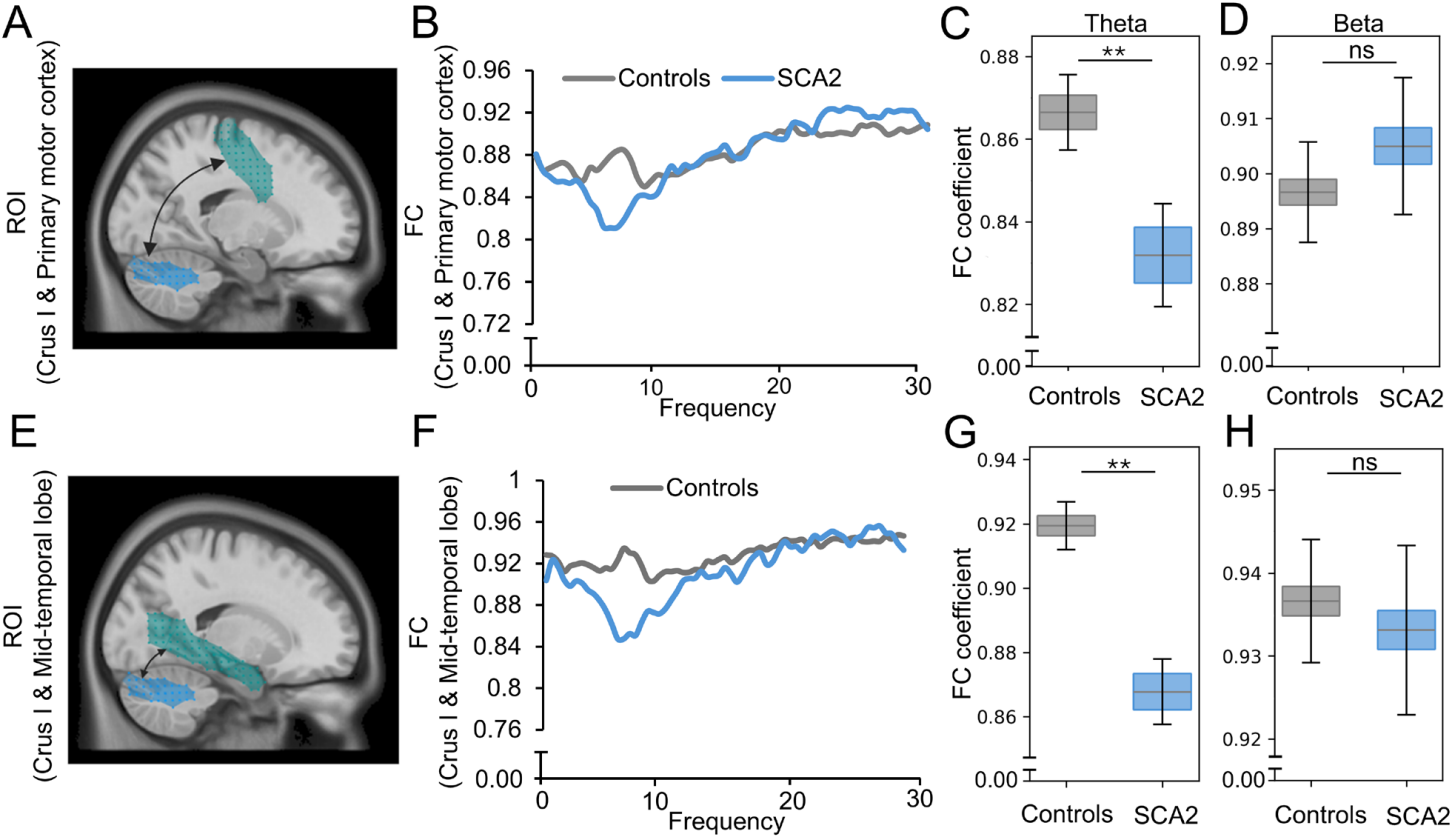
Functional connectivity between Crus I and primary motor cortex and mid‐temporal lobe. (A) Functional connectivity (FC) between Crus I and primary motor cortex. (B) FC spectral diagram between controls (*n* = 24) and SCA2 patients (*n* = 12) demonstrating (C) a significant decrease of FC in the theta frequency range and (D) no significant differences in beta frequency range. (E–H) FC between Crus I and mid‐temporal lobe. FC coefficient (*y*‐axis) demonstrates the mean and standard error from the functional connectivity values calculated for the respective cerebellar and prefrontal ROIs between controls and patients. **p < 0.01. ROI, region of interest.

To determine the specificity of these changes, we next investigated the functional connectivity between Crus I and the primary motor cortex or mid‐temporal lobe, two brain regions that are known not to have strong connectivity with Crus I of the cerebellum, as suggested by functional magnetic resonance imaging (fMRI) studies during resting state [37, 38, 39]. We selected the primary motor cortex (199 vertices, volume 19.52 cm^3^) and mid‐temporal lobe (268 vertices, volume 25.60 cm^3^) regions based on the AAL3 parcellations. Functional connectivity between Crus I and the primary motor cortex demonstrated a decrease in theta frequency (*p* = 0.001, Figure 3A–C) compared to controls, similar to the connectivity between Crus I and the prefrontal regions. On the other hand, SCA2 patients did not show any functional connectivity changes between Crus I and the primary motor cortex in the beta frequency range (Figure 3B,D) or in the delta frequency range (Figure S1C). We next calculated the functional connectivity between Crus I and the mid‐temporal lobe. This analysis demonstrated a decrease in theta (*p* = 0.001) in SCA2 patients, while beta frequency remained unchanged (Figure 3E–H). We additionally observed a reduction in the alpha frequency range in Crus I–mid‐temporal lobe connectivity, while delta frequency remained unchanged (Figure S1D). The *t*‐statistical values illustrating the functional connectivities between Crus I and the ROIs of the prefrontal cortex, primary motor cortex, and the mid‐temporal lobe are provided in Figure S2.

In summary, there are distinct alterations in the functional connectivity patterns of SCA2 patients between the cerebellum and prefrontal cortex, characterized by an increase in beta frequency connectivity. This increase in beta frequency connectivity is specific to cerebellum‐prefrontal cortex connectivity and is not observed in the cerebellum‐primary motor cortex or in the cerebellum‐mid‐temporal lobe. A decrease in theta frequency connectivity is less specific as this can be found in the connectivities between the cerebellum and all three cortical regions: prefrontal cortex, primary motor cortex, and mid‐temporal lobe.

### 3.4 Clinical Correlations

We next investigated the correlation between the functional connectivity observed in the beta frequency range between Crus I and the prefrontal cortex (superior frontal gyrus and orbitofrontal cortex) and cognitive performance in SCA2 patients, as measured by the CCAS scale.

**FIGURE 4:**
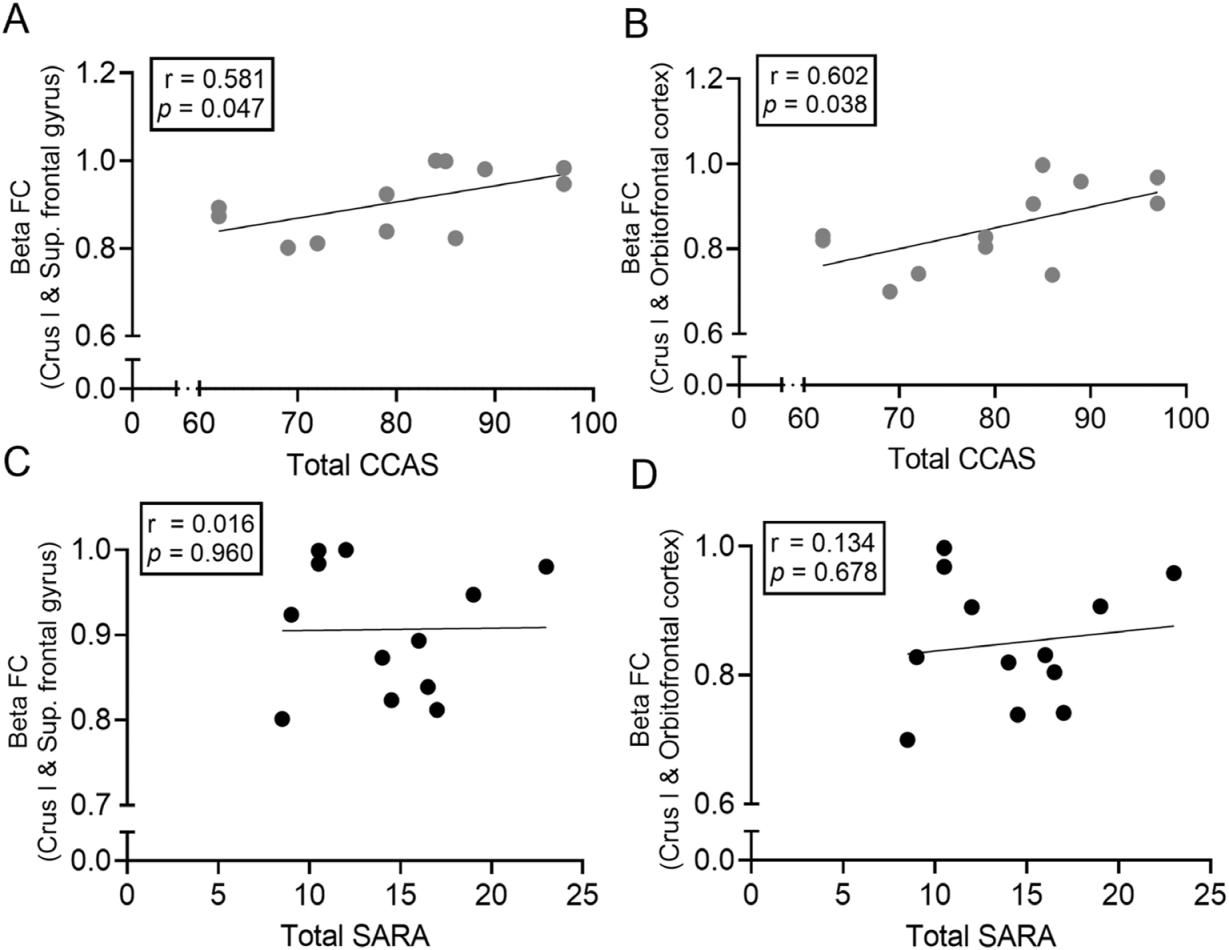
Correlation of functional connectivity scores with CCAS. Beta frequency functional connectivity scores for (A) Crus I and superior frontal gyrus, and (B) Crus I and orbitofrontal gyrus, significantly (*p* < 0.05) correlate with total CCAS scores. (C, D) However, the beta frequency functional connectivity scores did not correlate with the total SARA scores for these patients. CCAS, cerebellar cognitive affective syndrome; FC, functional connectivity; SARA, Scale for the Assessment and Rating of Ataxia; Sup, superior.

We found that there was a positive correlation between the total CCAS scores and the beta frequency functional connectivity for Crus I and the superior frontal gyrus (*r* = 0.581, *p* = 0.047, Figure 4A) and the orbitofrontal gyrus (*r* = 0.602, *p* = 0.038, Figure 4B), respectively. We next determined if the beta functional connectivity between Crus I and the prefrontal cortices is specific to the cognitive symptoms by studying the correlation with the motor symptoms. We found no correlation between the SARA scores and the beta frequency functional connectivity for Crus I and the superior frontal gyrus (*r* = 0.016, *p* = 0.960, Figure 4C) or the orbitofrontal gyrus (*r* = 0.134, *p* = 0.678, Figure 4D). Consistently, CCAS scores did not correlate with SARA scores, demonstrating that distinct circuit involvement of the motor and non‐motor cerebellum in SCA2. These results are consistent with the known function of the cognitive regions of the cerebellum (i.e., Crus I) and its connections to the prefrontal cortex.

## 4 Discussion

In this study, we investigated resting‐state activity using cerebellar‐cortical EEG to explore the functional connectivity between the cerebellum and prefrontal cortex as well as other parts of the cerebral cortex. We discovered a circuit dysfunction in the beta frequency of the cerebello‐prefrontal connectivity in SCA2 patients that correlates with cognitive impairment.

Resting‐state connectivity studies provide insights into cerebellar organization and its role in cognitive symptoms in SCA2 [40]. Previous fMRI studies on SCAs have reported connectivity changes linked to cognitive impairments. In SCA6, reduced attentional network connectivity correlated with executive and visual‐motor deficits [41]. In SCA3, reduced cerebellar‐cortical connectivity with somatomotor and frontoparietal networks, and increased dorsal attention network connectivity, were observed [42]. Similarly, SCA2 studies revealed reduced cerebellar network connectivity and increased default and frontoparietal network connectivity, correlating with motor and cognitive deficits [43]. However, these studies did not explore dysfunctional frequencies underlying these connectivity changes using EEG, which are critical for designing frequency‐specific neuromodulation strategies for therapeutic purposes.

The cerebellum modulates not only motor function but also cognition via the cerebello‐thalamo‐cortical circuits. Research reveals task‐specific activation patterns in the cerebellar regions, encompassing cognitive functions, particularly engaging posterior and lateral cerebellar regions including Crus I along with other posterior regions such as lobule VI, VII, and Crus II [44, 45]. Our study identified the most notable spectral differences in the source space in Crus I, making it the primary ROI for connectivity analysis. Dysfunctions in the posterior cerebellar region have been linked to the CCAS, which include executive function and emotional affect. By identifying the connectivity disruptions in beta frequencies, our findings provide insights into the circuit‐level dysfunction underlying these cognitive symptoms in SCA2, paving the way for neuromodulatory interventions.

Beta oscillations have been associated with attentional activation, sensorimotor integration, and the maintenance of ongoing cognitive operations, playing a role in top‐down control and decision‐making, particularly involving the prefrontal cortex [46]. Recent studies have shown that top‐down attentional modulation correlates with increased beta power and phase synchronization across the cerebral cortex, with growing evidence indicating that beta oscillatory activity reflects active inhibitory processes that help maintain the current cognitive and sensorimotor state by inhibiting oscillations in other frequency bands, as proposed by Engel and Fries [47, 48, 49]. Pathological enhancement of beta, as seen in SCA2 in our study, likely disrupts the brain's ability to flexibly shift cognitive control or process novel stimuli efficiently. In this context, the pathological enhancement of beta activity in SCA2 may reflect an imbalance in the cerebello‐cortical networks responsible for integrating and regulating cognitive functions. Similar increases in beta activity have been observed in other conditions, such as Parkinson's disease in the subthalamic nucleus [50]. Interestingly, we identified that the beta activity in the cerebellar system is related to the cognitive symptoms in SCA2, in contrast to the beta activity in Parkinson's disease that is related to the motor symptoms. These results may suggest beta activity in the cerebellar system is different from that in basal ganglia. Further in Alzheimer's, patients demonstrated that neurodegeneration predominantly impacted EEG metrics in the frontocentral regions, with increased high‐frequency oscillations (elevated beta and gamma power) and decreased low‐frequency oscillations (reduced delta power), suggesting initial compensatory mechanisms [25]. Additionally, a previous study has shown that beta power increases following psychosocial stress, reflecting top‐down attentional modulation that may act as a compensatory mechanism to redirect attentional resources toward the task at hand [51]. However, future longitudinal studies are required to better understand if this finding possibly reflects the compensatory mechanisms underplay and provides evidence of dynamic adaptation of the cerebellar circuit in the context of neurodegeneration in SCA2.

Our study has several limitations. Firstly, the sample size is relatively small, necessitating further investigation with a larger cohort in future studies. Future studies with larger cohorts should also explore subgroup comparisons across different categories of CCAS, focusing on the specific domains of task failures. It should also be considered that standardized performance‐based cognitive tests could complement the CCAS to provide a more detailed assessment of domain‐specific cognitive functions. Additionally, it is essential to recognize that EEG‐based deep source localization may pose challenges in pinpointing the exact sources, suggesting the potential need for alternative validation methods to address source‐based bias. Moreover, exploring connectivity with other cerebellar and prefrontal regions could provide insights into additional circuit‐related dysfunctions associated with cognitive symptoms. As a next step, imaging techniques such as fMRI or neurostimulation could be integrated with EEG to gain a deeper understanding of the underlying circuit dysfunction and to investigate whether beta activity is linked to compensatory mechanisms.

To date, there are only a few studies utilizing EEG to study human cerebellar circuits. Recent studies have shown that it is possible to detect cerebellar activity using surface EEG [14, 15, 16, 17, 18, 20, 52]. Our study further demonstrated that cerebello‐cortical EEG could be used as a tool to track circuit dysfunction in SCA2 patients, which may be extended to study other types of cerebellar ataxia in the future.

## 5 Conclusion

Our study identified that SCA2 patients have dysfunctional functional connectivity in the theta and beta frequency ranges, with beta frequency dysfunction being specific to cerebellar‐prefrontal connectivity and correlating with cognitive symptoms. Cerebello‐cortical EEG thus may serve as a tool to monitor circuit dysfunction associated with cognitive symptoms in SCA2, facilitating the development of targeted neuromodulation therapies.

## Author Contributions

Conceptualization: A.K., S.‐H.K. Methodology: A.K., M.‐K.P., S.‐H.K. Investigation: A.K., R.‐Y.L., Z.F., S.‐H.K. Visualization: A.K., Z.F., Y.L., S.‐H.K. Funding acquisition: M.‐K.P., S.‐H.K. Project administration: A.K., R.‐Y.L., Z.F., P.P., R.G. Supervision: M.‐K.P., S.‐H.K. Writing – original draft: A.K., S.‐H.K. Writing – review and editing: A.K., Z.F., P.P., R.G., M.‐K.P., S.‐H.K.

## Conflicts of Interest

The authors declare no conflicts of interest.

## Supporting information


**Figure S1.** Delta frequency range functional connectivity. Functional connectivity (FC) between (A) Crus I and superior frontal gyrus, (B) Crus I and orbitofrontal cortex, (C) Crus I and primary motor cortex, and (D) Crus I and mid‐temporal lobe did not demonstrate differences in delta frequency ranges. Sup, superior.

**Figure S2.** *T*‐statistical maps for functional connectivity. *T*‐values for functional connectivity between cerebellar (Crus I) and prefrontal ROIs (superior frontal gyrus, orbitofrontal cortex), as well as primary motor cortex and mid‐temporal lobe seen for (A) delta frequency, (B) theta frequency, and (C) beta frequency range. P, primary.

## Data Availability

The data that support the findings of this study are available from the corresponding author upon reasonable request.
