# Supplementary figures and images for: Cerebello‐Prefrontal Connectivity Underlying Cognitive Dysfunction in Spinocerebellar Ataxia Type 2
Source: Ann Clin Transl Neurol. 2025 Apr 3;12(6):1109–17. doi: 10.1002/acn3.70028 (PMC12172106; doi:10.1002/acn3.70028)

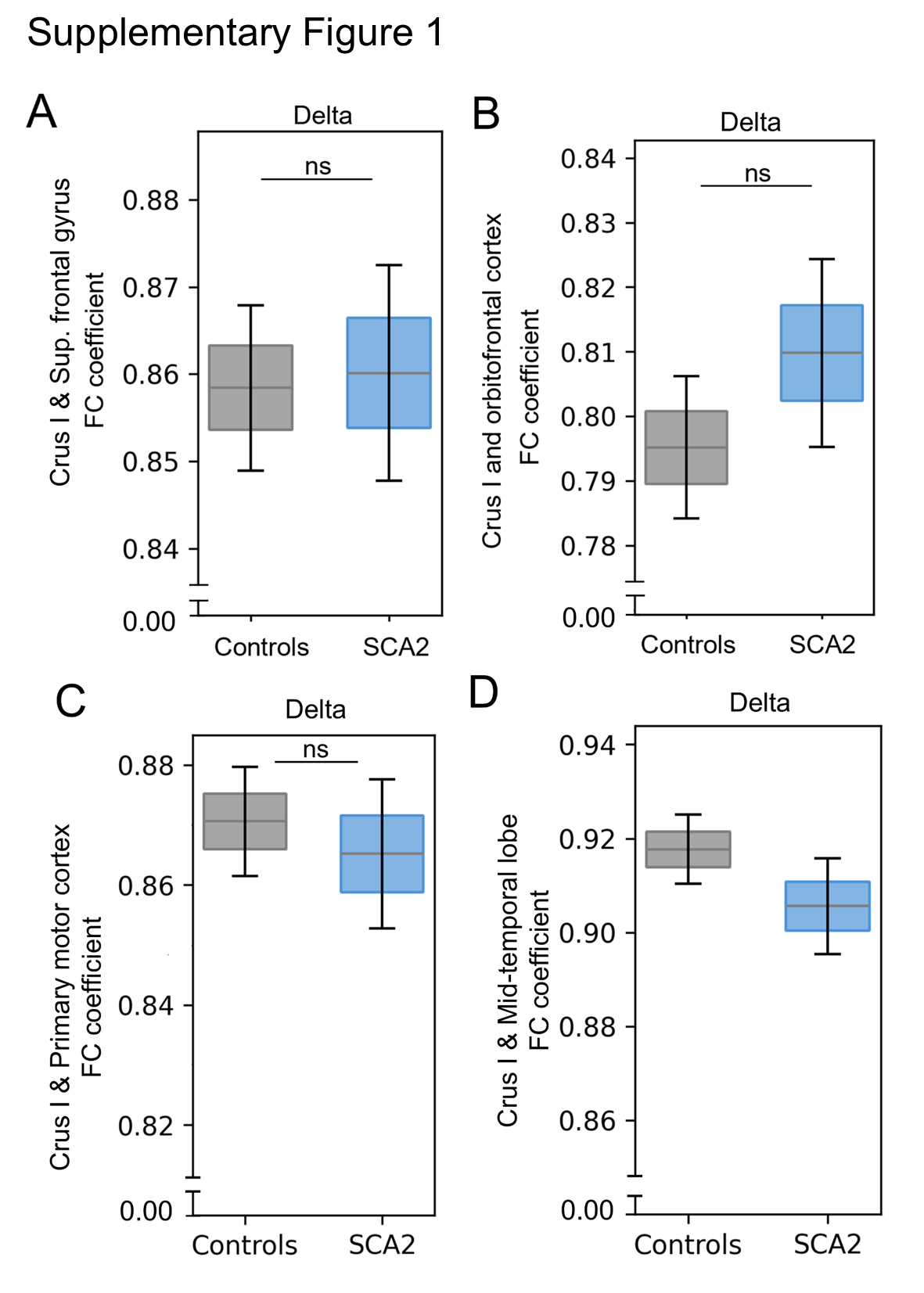

Supplement: Supplementary file 1 — Figure S1. Delta frequency range functional connectivity. Functional connectivity (FC) between (A) Crus I and superior frontal gyrus, (B) Crus I and orbitofrontal cortex, (C) Crus I and primary motor cortex, and (D) Crus I and mid‐temporal lobe did not demonstrate differences in delta frequency ranges. Sup, superior. [file ACN3-12-1109-s001.tif]

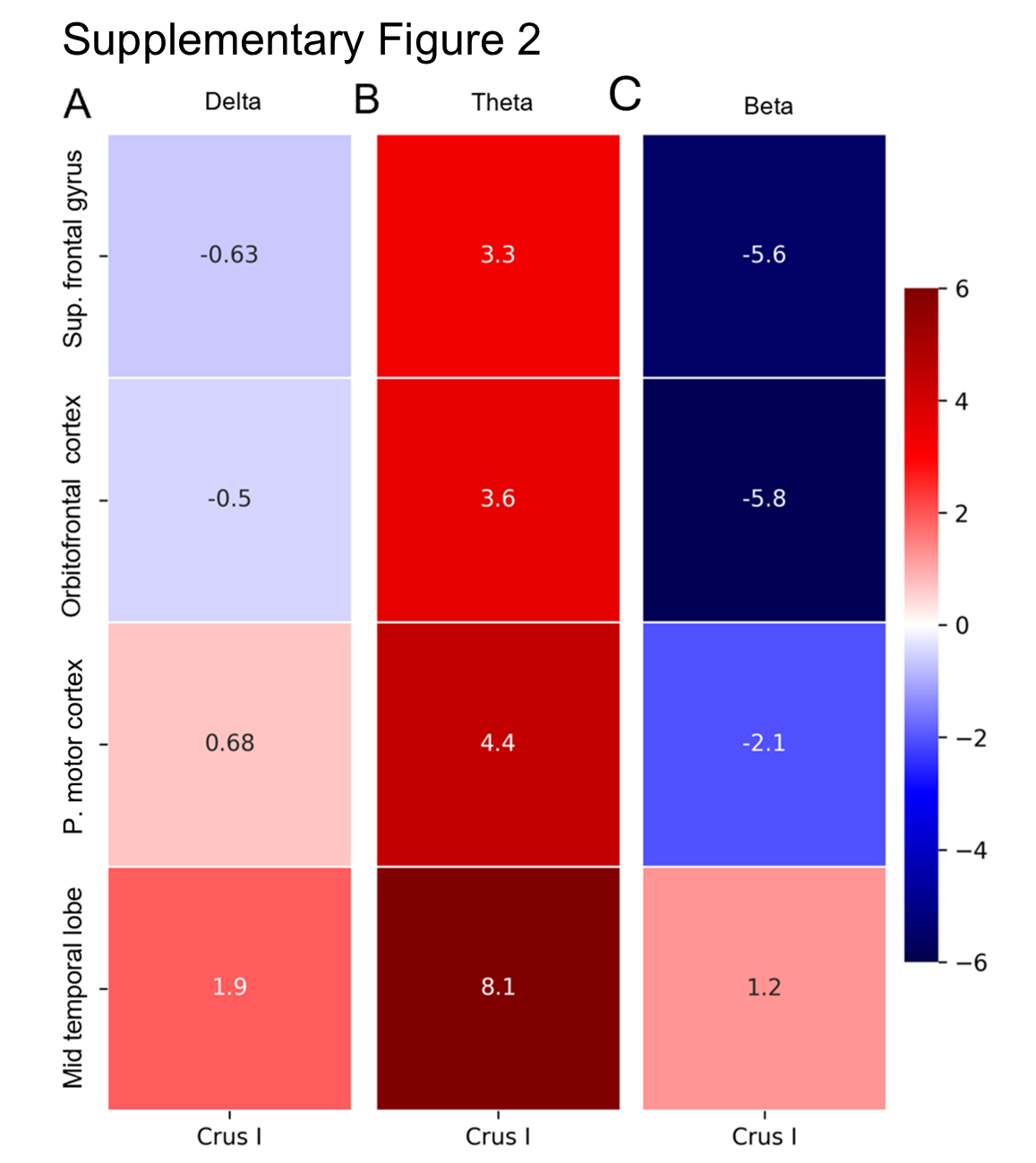

Supplement: Supplementary file 2 — Figure S2. T‐statistical maps for functional connectivity. T‐values for functional connectivity between cerebellar (Crus I) and prefrontal ROIs (superior frontal gyrus, orbitofrontal cortex), as well as primary motor cortex and mid‐temporal lobe seen for (A) delta frequency, (B) theta frequency, and (C) beta frequency range. P, primary. [file ACN3-12-1109-s002.tif]
